# Response to ‘T-helper 17 cell cytokines and interferon type I: partners in crime in systemic lupus erythematosus?’

**DOI:** 10.1186/ar4576

**Published:** 2014-06-09

**Authors:** Sebastian Dolff, Wayel H Abdulahad, Cees GM Kallenberg

**Affiliations:** 1 Department of Nephrology, University Hospital Essen, University Duisburg-Essen, Hufelandstr. 55, 45122 Essen, Germany; 2 Department of Rheumatology and Clinical Immunology, University Medical Center Groningen, University of Groningen, Hanzeplain 1, 9713 GZ, Groningen, The Netherlands

## 

We read with great interest the article by Brkic and colleagues in a recent issue of *Arthritis Research & Therapy*[1]. In that study, the authors investigated the distribution of T helper (Th) subsets which produce IL-17A, IL-17 F, IL-21, and IL-22 in patients with systemic lupus erythematosus (SLE) in relation to their genetic IFN type I signature. Patients with an IFN type I-positive signature showed increased percentages of IL-17A- and IL-21-producing CCR6^+^ T cells. From these results, the authors conclude that IFN type I cells co-act with Th17 cytokines in the pathogenesis of SLE. Surprisingly, they excluded CD25^+^ T cells from their analysis. In a previous study, we showed that Th cells from SLE patients expressing CD25^med^ and CD25^high^ are also able to produce IFN-γ and IL-17A [2]. Therefore, it would be relevant to assess cytokine expression in CD4^+^CD25^+^ T cells from IFN type I-positive and IFN type I-negative SLE patients. Furthermore, it should be proven that the genetic signature is solely responsible for the increased IFN production by Th cells. In addition, their finding that CCR6^+^ T cells are capable of producing IL-21 indirectly confirms our previous observation that IL-17^+^ T cells are a main source of IL-21 in patients with SLE [3]. Possibly, IL-21 is orchestrating the Th1/Th17 axis.

Finally, we agree that there might be a co-activity between IFN-I- and IL-17-producing cells as described by Brkic and colleagues. However, considering our findings that T cells with a regulatory phenotype are able to produce IFN-γ and IL-17A in patients with SLE, we suggest that primarily the plasticity of T cells is altered in patients with SLE [4].

## Abbreviations

IFN: Interferon; IL: Interleukin; SLE: Systemic lupus erythematosus; Th: T helper.

## Competing interests

The authors declare that they have no competing interests.

## Authors’ contributions

All authors contributed to the interpretation of data. SD drafted the manuscript. All authors read and approved the final manuscript.
